# Fleming Memorial Hospital, Newcastle-On-Tyne

**Published:** 1892-11-05

**Authors:** 


### FLEMING MEMORIAL HOSPITAL, NEWCASTLE-ON-TYNE.

We are glad to learn that the report of our Commissioner on the insanitary condition of this institution, which appeared in The Hospital of October 8th, p. 29, is likely to bear fruit. The honorary medical staff have very properly held more than one meeting, and they have now made a series of recommendations which we hope the governing body may decide to carry out without delay.

